# Transcriptional Silencing of Multiple Genes in Trophozoites of Entamoeba histolytica

**DOI:** 10.1371/journal.ppat.0020048

**Published:** 2006-05-26

**Authors:** Rivka Bracha, Yael Nuchamowitz, Michael Anbar, David Mirelman

**Affiliations:** Department of Biological Chemistry, Weizmann Institute of Science, Rehovot, Israel; Stanford University, United States of America

## Abstract

In a previous work we described the transcriptional silencing of the amoebapore A (AP-A) gene *(Ehap-a)* of Entamoeba histolytica strain HM-1:IMSS. The silencing occurred following transfection with a plasmid containing a 5′ upstream region (473 bp) of *Ehap-a* that included a truncated segment (140 bp) of a short interspersed nuclear element (SINE1). Silencing remained in effect even after removal of the plasmid (clone G3). Neither short interfering RNA nor methylated DNA were detected, but the chromatin domain of *Ehap-a* in the gene-silenced trophozoites was modified. Two other similar genes *(Ehap-b* and one encoding a Saposin-like protein, *SAPLIP 1)* also became silenced. In the present work we demonstrate the silencing of a second gene of choice, one that encodes the light subunit of the Gal/GalNAc inhibitable lectin *(Ehlgl1)* and the other, the cysteine proteinase 5 *(EhCP-5)*. This silencing occurred in G3 trophozoites transfected with a plasmid in which the 473 bp 5′ upstream *Ehap-a* fragment was directly ligated to the second gene. Transcriptional silencing occurred in both the transgene and the chromosomal gene. SINE1 sequences were essential, as was a direct connection between the *Ehap-a* upstream region and the beginning of the open reading frame of the second gene. Gene silencing did not occur in strain HM-1:IMSS with any of these plasmid constructs. The trophozoites with two silenced genes were virulence-attenuated as were those of clone G3. In addition, trophozoites not expressing Lgl1 and AP-A proteins had a significantly reduced ability to cap the Gal/GalNAc-lectin to the uroid region when incubated with antibodies against the heavy (170 kDa) subunit of the lectin. Lysates of trophozoites lacking cysteine proteinase 5 and AP-A proteins had 30% less cysteine proteinase activity than those of HM-1:IMSS strain or the G3 clone. Silencing of other genes in G3 amoebae could provide a model to study their various functions. In addition, double gene-silenced, virulence-attenuated trophozoites may be an important tool in vaccine development.

## Synopsis

The human intestinal parasite Entamoeba histolytica has numerous genes that code for virulence. Silencing the expression of individual genes is useful to determine their roles. In previous work the authors demonstrated the silencing of the gene coding for amoebapore, which is responsible for killing of human cells. They transfected amoebic trophozoites with a plasmid that contained DNA sequences homologous to the promoter region of the amoebapore gene, as well as a portion of a repetitive DNA element (called a short interspersed nuclear element). This construct induced a modification of the chromatin and prevented the expression of the gene. Removal of the plasmid resulted in stable, amoebapore-deficient parasites possessing low virulence. In the present work, Bracha and colleagues show silencing of additional genes following transfection of E. histolytica trophozoites already silenced in amoebapore with a plasmid containing the second gene directly ligated to the upstream region of the amoebapore gene. The DNA sequences that are essential for transferring the silencing from the plasmid to the chromosomal gene copy were identified. Additional virulence genes that the authors irreversibly silenced are those encoding a subunit of a surface lectin that mediates the adherence of the parasite to host cells, and a cysteine proteinase that plays a role in inflammation and invasion of the intestine.

## Introduction

Epigenetic gene silencing is a heritable change in gene expression that occurs without a change in nucleotide sequence. Homology-dependent silencing of gene expression has been reported in plants, animals, and fungi [1–7] and was shown to proceed by one of two mechanisms, inactivation at the transcriptional level (transcriptional gene silencing [TGS]) or at the posttranscriptional level (posttranscriptional gene silencing [PTGS]) [8,9]. TGS has been shown to occur in plants following transfection with plasmids containing a transgene promoter region without the transcribed sequences [6]. Suppression of expression was inheritable in the progeny and persisted even after the silencer sequence was excised [10]. In *Saccharomyces cerevisiae,* TGS was epigenetically maintained during many mitotic divisions by the formation of heterochromatin-like structures [11]. Epigenetic silencing of the Entamoeba histolytica amoebapore A gene *(Ehap-a)* occurred following transfection of trophozoites of virulent strain HM-1:IMSS with a hybrid plasmid containing a 5′ upstream region (473 bp) of the *Ehap-a* gene [12]. Nuclear run-on experiments showed that gene silencing was at the transcriptional level (TGS), and silencing persisted in the progeny even after removal of the plasmid [12]. Sequence analysis of the 473 bp upstream segment revealed that in addition to the promoter region of the *Ehap-a* gene, it included 140 bp of an adjacent, short interspersed nuclear element (SINE1) that is transcribed in the opposite orientation and also contained a unique thymidine-rich stretch of 48 bp. E. histolytica has been shown to harbor non-long terminal repeats that are either long interspersed (LINE) or SINEs [13–15]. These SINE1 repetitive elements, also termed IE/Ehapt2 [16,17], are noncoding retroposons that are widely dispersed in the E. histolytica genome and are abundantly transcribed. Recent findings suggest that LINE-encoded enzymes may play a role in SINE mobilization [15]. Other studies have shown that the transcriptional activity of some non-LTR transposable elements is sensitive to the presence of homologous transgenes and that the transcriptional activation of retrotransposons can alter the expression of adjacent genes in wheat as well as in mice [18–20].

We recently showed [21] that, in order to silence the *Ehap-a* gene, plasmids require the presence of at least 80 bp of the 5′ end of the SINE1 element, and that the transcription of the SINE1 element located directly upstream of the *Ehap-a* gene is significantly activated in the silenced trophozoites. The plasmidless *Ehap-a*-suppressed trophozoites of a clone termed G3 are phenotypically stable and have been maintained for over two years. All our attempts to restore the transcription of the *Ehap-a* gene, for example by growing the trophozoites in the presence of inhibitors of DNA methylation such as 5′ aza-cytidine or zebularine [22] or inhibitors of histone deacetylation such as trichostatin A [23] or sodium butyrate, have failed. Moreover, in contrast to trophozoites of parent strain HM-1:IMSS, retransfection of G3 trophozoites with a hybrid plasmid in which the *Ehap-a* gene was placed under the 5′ and 3′ regulatory elements of the ribosomal protein L21 gene *(EhRP-L21)* did not promote the expression of the amoebapore-A protein (AP-A) [12,24]. We also did not detect any short interfering RNA molecules (21–24 nucleotides) originating from the open reading frame (ORF) or the 5′ untranslated region (UTR) of the *Ehap-a* gene. This is not surprising, as neither Dicer nor Drosha RNase type III enzymes [25] were detected in the E. histolytica genome database [26]. RNA extracts from gene-silenced cultures revealed, however, the presence of small amounts of short (~140 nucleotides), single-stranded RNA molecules with homology to the 5′ end of the SINE element that is located upstream of the *Ehap-a* gene [21]. The molecular mechanism of the TGS discovered in E. histolytica is not yet completely clear, but it appears not to involve DNA methylation of the promoter region of the *Ehap-a* gene [12].We detected a modification in the chromatin of the silenced trophozoites, in the *Ehap-a* gene domain, whereby lysine 4 of histone H3 appears to be demethylated and does not precipitate with the anti-histone H3-methylated-Lys4 in a chromatin immunoprecipitation experiment [21]. In other systems the demethylation of H3Lys4 has been shown to correspond with transcription-inactive domains [27–29]. Unfortunately, due to considerable sequence divergence between the histones of E. histolytica and those of higher organisms [30], we have not yet been able to determine whether there are additional histone modifications in the silenced amoebae. Our results suggest the involvement of the truncated SINE1 element in triggering the gene silencing, possibly by the single-stranded RNA molecules that originated from it, and a role for histone modification enzymes in its epigenetic maintenance as has been proposed [31–33].

In order to further investigate the gene-silencing capabilities of E. histolytica trophozoites, we decided to silence additional virulence genes of interest. This was achieved by cloning the ORF of a second gene under the control of the 5′ upstream region (473 bp) of the *Ehap-a* gene and transfecting the hybrid plasmids into the already silenced G3 trophozoites. Using this approach we succeeded in silencing several genes, among them those encoding the light subunit of the Gal/GalNac inhibitable lectin, *Ehlgl1* [34], and cysteine proteinase 5, *EhCP-5,* both of which are important virulent factors of the parasite [35–42]. Their lack of transcription was complete both from the transgene as well as from the endogenous genomic copy. Interestingly, silencing of the second gene did not occur in trophozoites of parent strain HM-1:IMSS transfected with the above plasmids, whereas overexpression of the gene was observed.

We thus report here on the generation of two new trophozoite clones each silenced in two different important virulence genes whose silencing remained in effect even after removal of the plasmids. In addition, we determined the different regions of the 5′ upstream sequence of the *Ehap-a* gene that are important for the silencing of a second gene. The molecular marker and the cellular machinery by which the heterochromatin spreads, in trans, to the genomic copy of the second gene is not yet clear and is under intense investigation in numerous laboratories [31–33,43]. Our novel method of silencing of other genes of interest in the *Ehap-a-*silenced trophozoites can serve as a tool to further understand the role and function of certain genes of the parasite. The method may also be useful for the production of E. histolytica cultures silenced in multiple virulence genes that could perhaps serve in the search for a vaccine.

## Results

### Changes in the Transcription of Other Genes during the Epigenetic Silencing of the *Ehap-a* Gene

**Figure 1:**
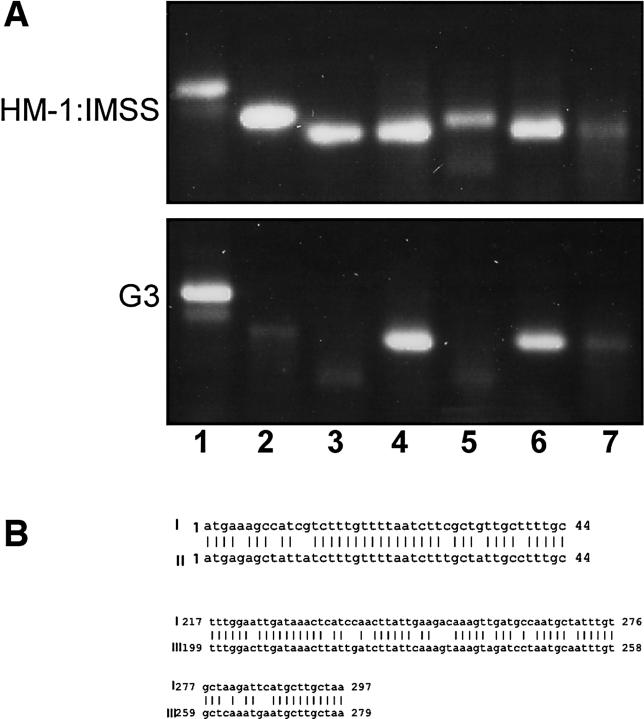
Changes in the Transcription of Other Amoebapore-Like Genes in G3 Trophozoites as Determined by RT-PCR (A) Total RNA was prepared from freshly harvested HM1-IMSS and G3 trophozoites. The RNA was treated with RNase-free DNase, and reversed transcribed using oligo dT-adaptor primer (Table 1, primer I). PCR was then performed using a sense primer from the gene of interest (Table 1) and the adaptor primer (Table 1, primer II) as antisense for all the genes. Lanes: 1, *EhRPL21;* 2, *Ehap-a;* 3, *Ehap-b;* 4, *Ehap-c;* 5, *SAPLIP 1*; 6, *SAPLIP 5*; 7, *SAPLIP 14*. (B) Sequence comparisons between the three genes that were silenced: I, *Ehap-a;* II, *Ehap-b;* III, *SAPLIP 1*.

In previous work [12,44] we demonstrated that, in addition to *Ehap-a,* in G3 trophozoites the gene coding for amoeobapore B *(Ehap-b)* was also epigenetically silenced in the plasmidless G3 trophozoites. Another gene, which we found was silenced in G3 trophozoites, codes for a saposin-like protein (SAPLIP 1) [45,46]. The down-regulation of the SAPLIP 1-encoding gene was first noted by Dr. Upi Singh (Stanford University, United States), following hybridizations of genomic microarrays with cDNA produced from mRNA of G3 trophozoites as compared to that of HM-1:IMSS trophozoites (U. Singh, personal communication). The transcription levels of various *SAPLIPs* as well as other housekeeping genes was examined by RT-PCR, and as shown in Figure 1A the transcripts of *Ehap-a, Ehap-b,* and *SAPLIP 1* were down-regulated in the silenced G3 trophozoites, while others, such as *Ehap-c, SAPLIP 5,* and *SAPLIP 14* showed transcription levels simliar to those in the parent strain HM-1:IMSS. Similar levels of transcription were also detected for the ribosomal protein gene *EhRP-L21* and the gene encoding E. histolytica actin *(Ehactin)* (unpublished data).

**Table 1:**
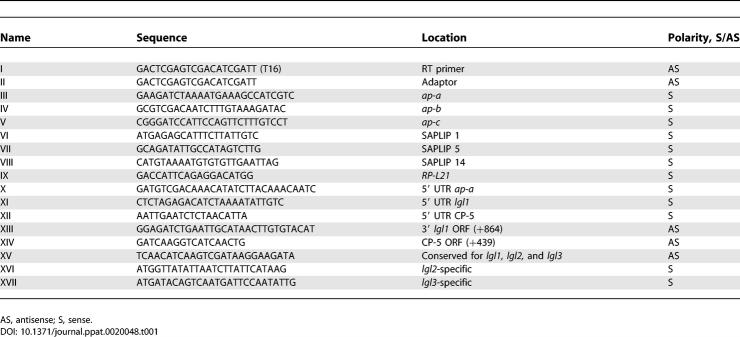
Primers Used for RT-PCR

Considerable sequence homology was found between *Ehap-a, Ehap-b,* and *SAPLIP 1* (Figure 1B) [45,46]. According to the phylogenetic tree of SAPLIPs and amoebapore proteins [45,46], *Ehap-b* and *SAPLIP 1* are on the same branch as *Ehap-a*. This homology was restricted to the ORF region of those genes. No homology was found in the regulatory sequences, and SINE1 sequences are not present upstream to those genes. Furthermore, the two nonsilenced *SAPLIP* genes tested *(SAPLIPs 5* and *14),* as well as *Ehap-c* (Figure 1A), have no significant sequence homology to *Ehap-a.*

### Silencing of Other Virulence Genes in Plasmidless Silenced G3 Trophozoites

#### The light subunit of the Gal/GalNAc lectin *(Ehlgl1)* gene.

**Figure 2:**
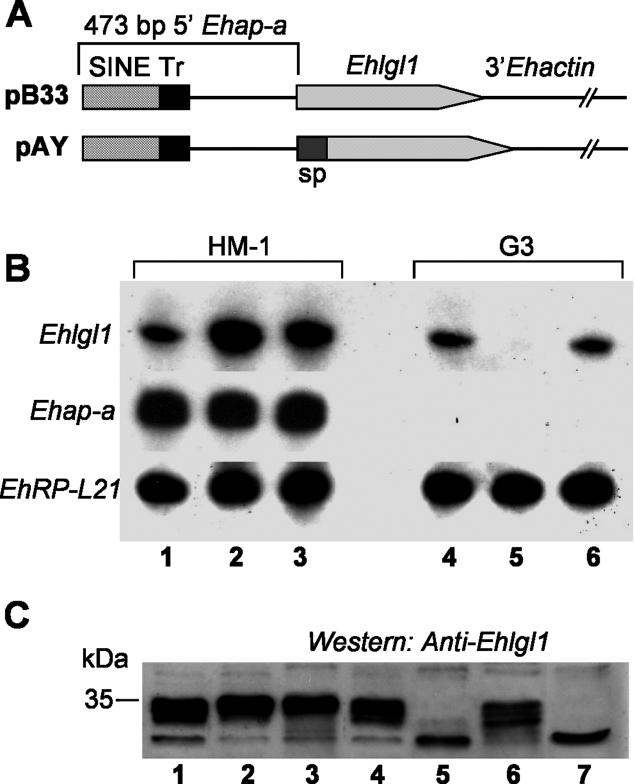
Silencing of the *Ehlgl1* Gene (A) Diagrams of the *Ehlgl1*-containing plasmids pB33 and pAY. The 473 bp *Ehap-a* promoter, SINE and T-rich (Tr) regions are marked. Plasmid pAY includes 44 bp of the signal peptide (sp) of the *Ehap-a* gene. Both plasmids contain the ORF of *Ehlgl1* gene and the 3′ regulatory sequence from the *Ehactin* gene. (B) Northern blot analysis of amoebic RNA extracts. Lanes: 1, untransfected HM1:IMSS; 2, HM-1:IMSS transfected with plasmid pB33; 3, HM-1:IMSS transfected with pAY; 4, untransfected clone G3; 5, clone G3 transfected with pB33; 6, clone G3 transfected with pAY. Blots were probed as indicated. (C) Western blot analysis of protein lysates separated on SDS-PAGE (12%) showing the blot that reacted with the polyclonal antibodies against the Lgl1 protein. Lanes 1–6 are as (B). Lane 7 contains lysate from the plasmidless RBV trophozoites (see Results).

In a previous study we showed that the Lgl1 protein plays a role in E. histolytica virulence [35,47]. In order to determine whether genes other than *Ehap-a* could be transcriptionally silenced by targeting the spreading of the silencing machinery from the already silenced sequences to other genes of interest, two plasmids containing the *Ehlgl1* gene were constructed (Figure 2A). In the first plasmid (pB33) we ligated the entire ORF of the *Ehlgl1* gene to the 473 bp of the 5′ upstream sequence of the *Ehap-a* gene and the 3′ regulatory sequence of *Ehactin*. In the second plasmid (pAY) 44 bp of the *Ehap-a* gene signal peptide (“sp” in Figure 2A) [48] was added to the 3' end of the 473 bp fragment and ligated to the ORF of the *Ehlgl1* gene.This 44 bp segment was highly homologous to that of the *Ehap-b* gene, which, as mentioned above (Figure 1A and 1B), was also silenced in G3. The rest of the pAY construct, the *Ehlgl1* ORF and 3′ *Ehactin,* was identical to pB33. The two plasmids were transfected into trophozoites of HM-1:IMSS and G3, and RNA isolated from the four transfectants was analysed by Northern blot (Figure 2B).

In G3 trophozoites, a marked down-regulation occurred in the expression of *Ehlgl1* in the pB33 transfectants; no such effect was seen in the pAY transfectants. In the parent strain, HM-1:IMSS, an up-regulation was seen in the level of *Ehlgl1* transcript in both pB33 and pAY transfectants (Figure 2B), which indicates that the plasmid expressed the downstream gene. The level of ribosomal protein *EhRP-L21* transcript served as loading control and was similar in all cultures. The *Ehap-a* gene transcript was totally absent in G3-derived samples but was equally expressed in all the transfected HM-1:IMSS cultures. Western blots of the above samples revealed a similar picture (Figure 2C). Lgl1 protein (35 KDa) was not observed in G3 transfected with pB33 plasmid nor in the plasmidless RBV culture (pB33-derived, silenced in *Ehap-a* and *Ehlgl1;* see below) but was present in all other cultures. An unidentified lower band that cross-reacted with the anti-Lgl1 antibodies appears in all the samples. The EhAP-A protein, as expected, was absent from cultures derived from G3 but present in all the HM-1:IMSS transfected cultures (unpublished data).

#### The source of the *Ehlgl1* transcript in the different transfectants.

**Figure 3:**
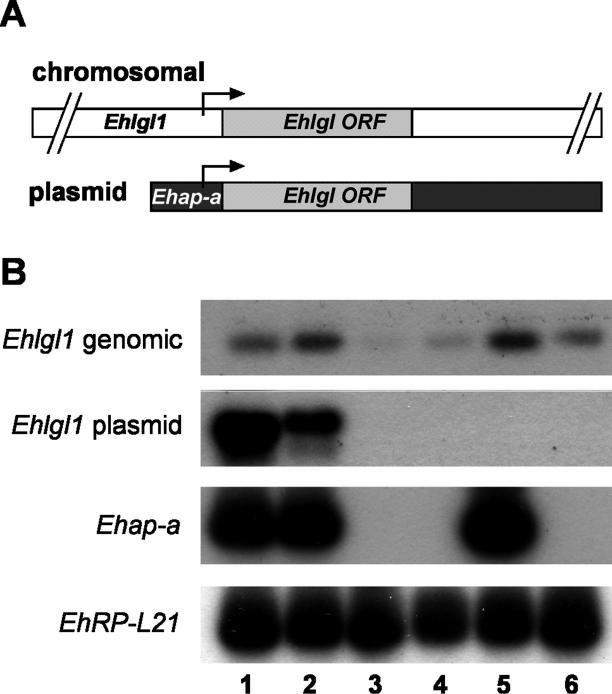
Identification of the Source of *Ehlgl1* Transcripts (A) Diagrams of the location of the two different sense primers used to differentiate between the two types of *Ehlgl1* transcripts (genomic and plasmid-derived). (B) RT-PCR analysis of different transfectants. Isolated RNA was subjected to reverse transcription and PCR for several types of transcripts: genomic *Ehlgl1* (Table 1, primers XI + XIII), *Ehlgl1* derived from plasmid (primers X + XIII), *Ehap-a* (primers II + III), and *EhRP-L21* (primers II + IX). Lanes: 1, HM-1:IMSS transfected with pB33; 2, HM-1:IMSS transfected with pAY; 3, G3 transfected with pB33; 4, G3 transfected with pAY; 5, untransfected HM-1:IMSS; 6, untransfected G3.

Since the *Ehlgl1* chromosomal gene copy contains the sequences of the *Ehlgl1* UTR while the transgene *Ehlgl1* copy contains sequences of the *Ehap-a* UTR*,* mRNA from the pB33 and pAY transfectants can be transcribed from two sources. RT-PCR performed on the respective mRNA samples using primers designed for the different UTRs (Figure 3A and Table 1) revealed that G3 cultures transfected with pB33 plasmid had no *Ehlgl1* transcripts from the chromosomal gene nor from the plasmid copy (Figure 3B). Trophozoites of parent strain HM-1:IMSS transfected with pB33 showed high *Ehlgl1* transcription from the transgene copy of the gene, and levels from the chromosomal copy similar to the nontransfected control (Figure 3B). G3 trophozoites transfected with plasmid pAY revealed that the transcript of *Ehlgl1* (Figure 2B) originated from the chromosomal gene copy, whereas the transgene copy was not transcribed and remained silenced. HM-1:IMSS transfected with pAY also showed transcription of *Ehlgl1* from both the transgene and the chromosomal copy (Figure 3B). A similar level of ribosomal protein L21 (gene *EhRP-L21*) RT-PCR product was observed in all cultures, and the *Ehap-a* transcript was absent from all G3-derived RNA, as seen on Northern blots (Figure 2B).

The difference between the results obtained with pB33 and pAY in G3 transfectants indicates that the ability to spread the silencing to an additional chromosomal gene in trans from the already silenced promoter of the *Ehap-a* gene required the direct ligation of the *Ehlgl1* ORF to the 473 bp 5′ upstream region of *Ehap-a* gene, as was the case with plasmid pB33. In plasmid pAY, which had an interruption due to the introduction of a 44 bp sequence of the *Ehap-a* gene ORF, only the *Ehlgl1* from the episomal gene was silenced; the chromosomal gene remained active.

#### Phenotype of the trophozoite cultures silenced in *Ehap-a* and *Ehlgl1*.

**Figure 4:**
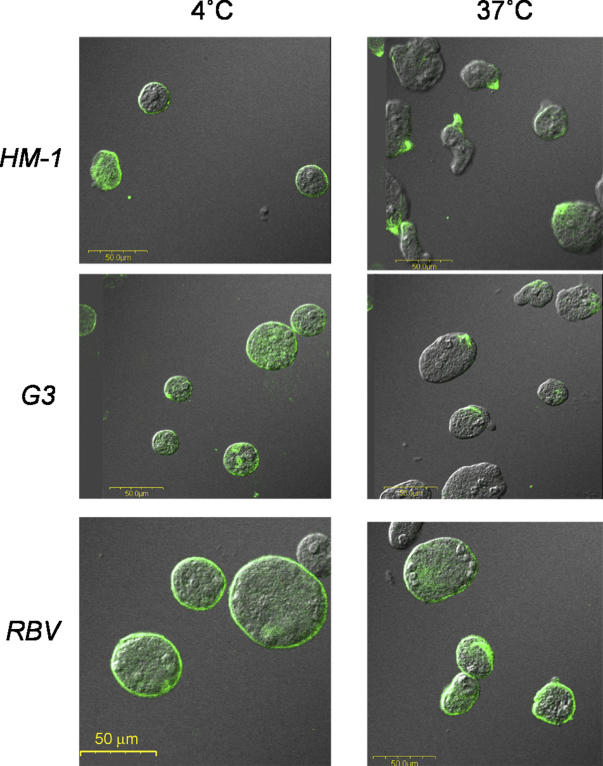
Induction of Capping of the Gal/GalNAc-Lectin to the Uroid Region of the Trophozoites Confocal microscopy of trophozoites; left photomicrographs, trophozoites incubated at 4 °C; right photomicrographs, trophozoites were incubated at 37 °C for 20 min to observe the induction of capping using two monoclonal antibodies against the heavy subunit of the Gal-lectin [35]. Each image shows the fluorescent Gal-lectin superimposed on a Nomarsky section. Samples are HM-1:IMSS, G3, and RBV (the plasmidless strain silenced in *Ehlgl1* and *Ehap-a*).

In order to find a specific effect of silencing of the *Ehlgl1* gene, we examined the ability of the plasmidless RBV trophozoites silenced in the two genes (see below) to perform cell capping of a surface antigen. We had earlier shown that transfectants overexpressing a dominant-negative N-truncated Lgl1 were defective in their ability to cap the surface Gal/GalNAc-specific lectin molecules to the uroid region of the amoeba [35]. A very similar result was observed with the plasmidless, double-silenced RBV trophozoites, which are completely devoid of Lgl1 protein. As shown in Figure 4, RBV trophozoites were unable to cap the Gal/GalNAc lectin molecules following their interaction with monoclonal antibodies against the heavy subunit of the lectin molecules, whereas capping was clearly observed in G3 and HM-1:IMSS trophozoites.

#### Silencing of the cysteine proteinase 5 gene, *EhCP-5*.

**Figure 5:**
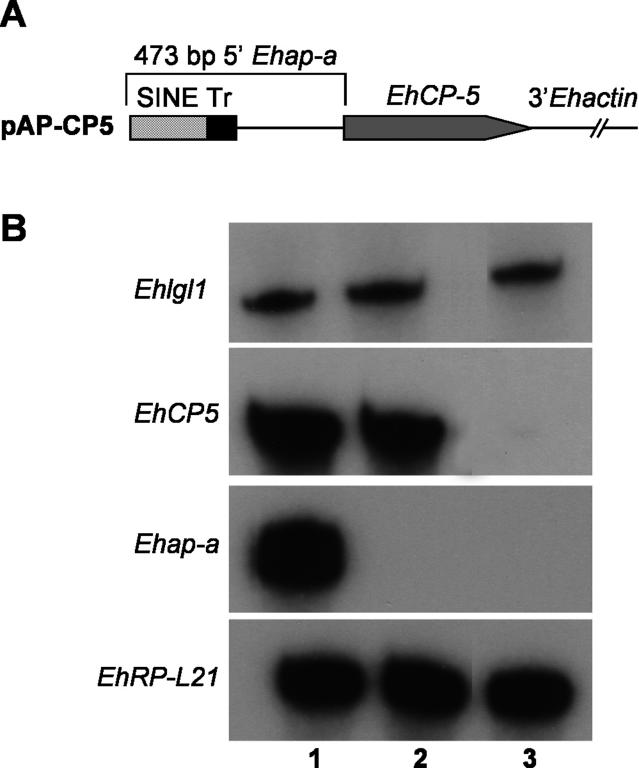
Silencing of Cysteine Proteinase 5 *(EhCP5)* (A) Diagram of the pAP-CP5 construct. (B) Northern blot analysis. Lanes: 1, RNA from untransfected HM1:IMSS; 2, RNA from untransfected G3; 3, RNA from G3 transfected with pAP-CP5 (probes used are as indicated).

**Figure 6:**
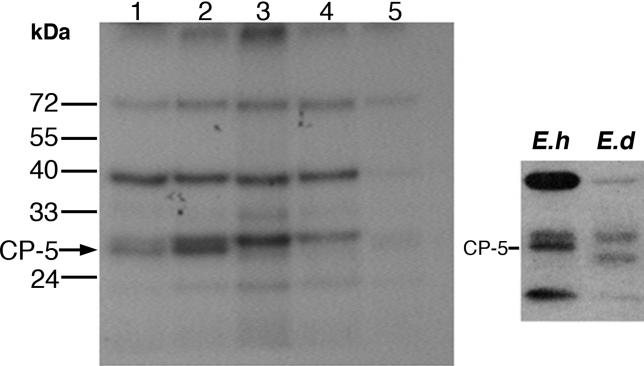
In Vivo Labeling of Cysteine Proteinases The different trophozoite cultures were grown for 18 h with the radiolabeled cysteine proteinase inhibitor Fmoc-[I^125^]Tyr-Ala-diazomethylketone (10 μg/ml, 10 μCi/ml), harvested, washed, and lysed [49]. Samples of each cell lysate (25 μg) were separated on a 12% acrylamide reducing gel and exposed to X-ray film to reveal the different cysteine proteinase bands. Samples in the lanes on the left radiograph are: 1, untransfected HM-1:IMSS; 2, untransfected G3; 3, G3 transfected with pAP-CP5 and grown with 50 μg/ml of G418; 4, RB8 trophozoites after removal of the plasmid (see Results); 5, HM-1:IMSS grown with a lower specific activity inhibitor (0.05 μCi/μg). Samples on the right radiograph demonstrate the CP-5 band location in HM-1:IMSS and its absence from the E. dispar culture [50].

Cysteine proteinase 5 has been reported to be one of the most important virulence factors of E. histolytica [36–42]. In order to ascertain that it is possible to silence an additional gene using the same principle as that applied to the silencing of *Ehlgl1,* we constructed a plasmid, pAP-CP5, analogous to the pB33 plasmid (Figure 2A); however, instead of the *Ehlgl1* ORF, we introduced the ORF of the *EhCP-5* gene (Figure 5A). Transfection with plasmid pAP-CP5 of clone G3 trophozoites resulted in total silencing of the transcription of the *EhCP-5* gene as revealed by Northern blot (Figure 5B): both the endogenous gene and the transgene were silenced. The lack of expression of the CP-5 enzyme was also demonstrated by labeling the cysteine proteinases of the trophozoites in vivo with Fmoc-[I^125^]-Tyr-Ala-diazomethylketone, a specific inhibitor of cysteine proteinases that binds covalently to such enzymes [49]. The labeling results (Figure 6) demonstrate the disappearance of the CP-5 band in the pAP-CP5-transfected culture as well as from a plasmidless culture, RB8 (pAP-CP5-derived, silenced in *Ehap-a* and *EhCP-5;* see below). In both HM-1:IMSS and clone G3 the bands corresponding to CP-5 were detected (Figure 6, left). The result seen with the CP-5-deficient culture is similar to that obtained from trophozoites of the avirulent strain *E. dispar,* which is known to be devoid of CP-5 (Figure 6, right) [36,50]. The total cysteine proteinase activity detected in trophozoite lysates of the AP-A- and CP-5-deficient, plasmidless RB8 trophozoites was approximately 30% less than that in lysates of G3 or HM-1:IMSS trophozoites (determined spectrophotometrically, in units per mg of protein, from the degradation of the cysteine proteinase-specific substrate Z-Arg-Arg-pNA) (unpublished data). This result correlates well with the similar level of active bands of other cysteine proteinases seen in lysates of the CP-5-deficient trophozoites (Figure 6).

### The Double Gene-Silenced Cultures Remain Silenced after Removal of the Plasmids

**Figure 7:**
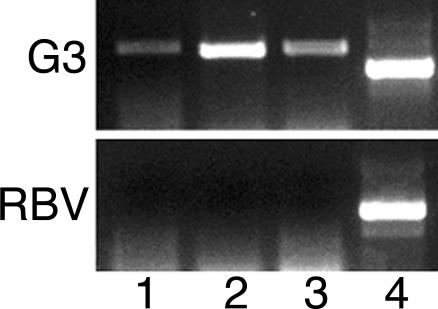
Determination of Transcripts of the Three Different *Ehlgl* Genes by RT PCR Total RNA was prepared from freshly harvested G3 and RBV trophozoites. PCR was performed using antisense conserved primer for *Ehlgl1, Ehlgl2, and Ehlgl3* (Table 1, primer XV) and a specific sense primer for each of the genes as indicated. Lanes: 1, primer specific for *Ehlgl1* (Table 1, primer XI); 2, primer specific for *Ehlgl2* (Table 1, primer XVI); 3, primer specific for *Ehlgl3* (Table 1, primer XVII); 4, primers specific for *EhRPL21* (Table 1 primers II + IX).

The removal of plasmids pB33 and pAP-CP5 from the double gene-silenced cultures was similar to how the G3 culture was generated [12]. The double-silenced trophozoites were cultured for more than 30 generations without the selective drug G418, then single trophozoites were cloned. Cultures were grown from the clones that were devoid of the neomycin resistance gene *(Neo).* The plasmidless cultures were silenced in two genes each, *Ehap-a* and *Ehlgl1,* in the pB33-derived trophozoites (called RBV) (Figure 2C) and *Ehap-a* and *EhCP-5* in the pAP-CP5-derived trophozoites (RB8) (Figure 6). In addition, we observed that in RBV trophozoites, no transcription of the two additional Gal/GalNAc lectin light subunit genes *Ehlgl2* and *Ehlgl3* occurred (Figure 7). As previously shown for G3 trophozoites [12], Southern blot restriction fragment-length polymorphisms, and comparisons of the sequences of the genomic DNA upstream and downstream of the silenced genes with that of the parent strain, showed no differences, indicating that no integration of plasmid sequences occurred in the genome (unpublished data).

### Virulence of Double Gene-Silenced Cultures

As previously reported [12,44,51], the G3 culture was avirulent in many of the assays used to evaluate virulence. Since the double gene-silenced, plasmidless trophozoites RBV and RB8 are derived from the plasmidless *Ehap-a* silenced clone G3, it was reasonable to assume that RBV or RB8 trophozoites would also possess low virulence. In contrast to trophozoites of strain HM-1:IMSS, which caused very large liver abscesses in Syrian golden hamsters (four animals), the three types of silenced amoebae (G3, RBV, and RB8) were incapable of inducing hepatic lesions following injection of 5 × 10^5^ trophozoites per liver in four animals tested for each strain.

### Sequences Required to Spread Gene Silencing to a Second Gene

**Figure 8:**
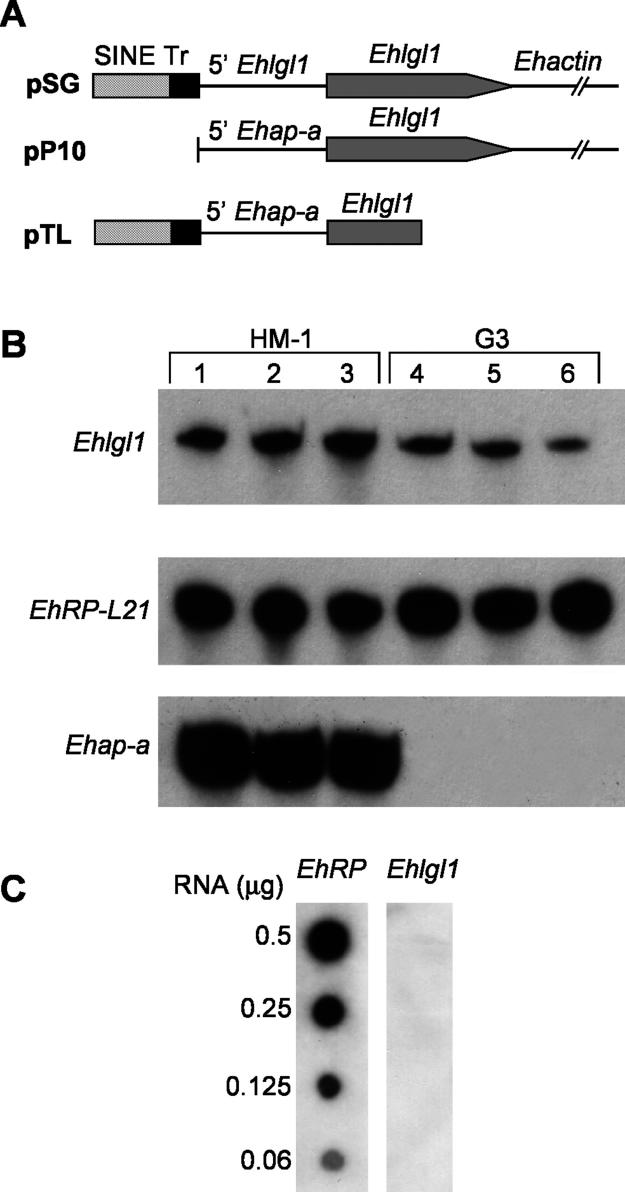
Additional Plasmid Constructs Tested for Their Silencing Capabilities (A) Diagrams of plasmid pSG in which the truncated SINE 1 and the T-rich sequences were ligated upstream to the *Ehlgl1* promoter and ORF regions. Plasmid pP10 has the SINE1 sequences removed from the *Ehap-a* promoter region. Plasmid pTL contains a truncated segment of the *Ehlgl1* gene without the 3′ regulatory region. Plasmid constructs can be compared to plasmid pB33 in Figure 2. (B) Northern blots of RNA extracts of trophozoite transfectants. Probes used as indicated. Lanes: 1, untransfected HM-1:IMSS; 2, HM-1:IMSS transfected with pSG; 3, HM-1:IMSS transfected with pP10; 4, G3; 5, G3 transfected with pSG; 6, G3 transfected with pP10. (C) Dot blot of RNA extracts from G3 trophozoites transfected with plasmid pTL and hybridized with probes for either *EhRP-L21* or *Ehlgl1.*

We have shown that in order to silence the *Ehap-a* gene, the plasmid must contain, in addition to the 5′ upstream region of the *Ehap-a* gene, a truncated segment (>80 bp) of a SINE1 repetitive element that is situated at the 5′ end of the *Ehap-a* regulatory region [21]. Omission of the truncated sequences of the SINE1 element resulted in overexpression of the *Ehap-a* gene. To find out which are the sequences required for the silencing of the second gene, we constructed two additional plasmids each carrying different regions of the 473 bp 5′ upstream flanking region of the *Ehap-a* gene. In the first plasmid, pSG (Figure 8A), 195 bp of the truncated SINE1 and the T-rich region that precedes it [21] were ligated to 300 bp of the 5′ upstream region of the *Ehlgl1* gene followed by the *Ehlgl1* ORF and the 3′ regulatory sequence of the *Ehactin* gene. The second plasmid, pP10 (Figure 8A), lacked the sequences of the SINE1 element and contained only 275 bp of the 5′ upstream region of *Ehap-a* fused to the *Ehlgl1* gene as above. This construct is somewhat similar to another plasmid, called psAP-10, which promoted overexpression of the *Ehap-a* gene and did not cause silencing when transfected into parent strain HM-1:IMSS [21]. Both plasmids, pSG and pP10, were transfected into both HM-1:IMSS and clone G3 trophozoites. The results from Northern blots (Figure 8B) and from RT-PCR experiments on total RNA extracted from the transfectants indicate that *Ehlgl1* is not silenced in either of the two transfected cultures. RNA extracts from pP10 transfectants of both HM-1:IMSS and clone G3 revealed that *Ehlgl1* transcripts were produced from both the plasmid-encoded *Ehlgl1* gene, as revealed by RT-PCR using primers X and XIII (Table 1) and the chromosomal copy, which remained at a similar level as the control (unpublished data). The conclusion from these different transfectants as well as from those shown in Figures 2 and 5 is that (i) the presence of both the SINE truncated sequences and the proximal sequences of the 5′ upstream region of the *Ehap-a* gene are essential for the silencing of the second gene, and (ii) the first ATG start codon of the second gene must be placed directly at the 3′ end of the 473 bp of the 5′ upstream fragment.

### Is the Entire ORF of the Second Gene Needed to Induce Silencing?

The transcription initiation site of a gene usually contains sequences from the 5′ UTR as well as sequences from the 5′ region of the ORF. This region is crucial for induction or inhibition of transcription. The question we posed was whether, to silence the chromosomal copy of the second gene, it is enough to introduce only a truncated ORF starting at its start codon and without any 3′ regulatory sequences. This of course could ease the process of silencing long genes. G3 trophozoites were transfected with a plasmid, pTL (Figure 8A), in which the 473 bp fragment of the 5′ upstream region of the *Ehap-a* gene was ligated to a truncated *Ehlgl1* fragment of 421 bp (+1 to +421) without the 3′ *Ehactin* regulatory sequences. Silencing of the chromosomal *Ehlgl1* gene was successful in the pTL transfectants, as shown by the complete absence of transcripts of *Ehlgl1* in RNA dot blots (Figure 8C) and from RT-PCR results (unpublished data) indicating that silencing of a second gene can be accomplished with a truncated second gene and in the absence of a 3′ regulatory region.

### Clone G3 Trophozoites Are Required for Silencing of a Second Gene

As described above, the successful silencing of a second gene *(Ehlg1l)* was achieved by transfection with plasmid pB33 of the plasmidless G3 trophozoites. Clone G3 trophozoites derive from silencing with plasmid psAP-2, which contained only 473 bp of the 5′ upstream region of the *Ehap-a* gene, and clone F1 trophozoites derive from silencing with plasmid psAP-1, which also included the ORF and the 3′ region of the *Ehap-a* gene [12]. Attempts to silence the *Ehlgl1* gene by transfection with plasmid pB33 of the plasmidless trophozoites of clone F1, failed. Transfectants of clone F1 trophozoites with plasmid pB33 remained silenced in the *Ehap-a* gene but were not silenced in the *Ehlgl1* gene (unpublished data) in contrast to transfectants of clone G3 shown above (Figure 2).

### Is It Possible to Silence More than Two Genes Successively?

Following the removal of the plasmid pB33 from the G3 transfected culture and generation of the plasmidless, *Ehap-a* and *Ehlgl1* gene-silenced RBV trophozoite culture, we retransfected them with pAP-CP5 (Figure 5A) to examine the possibility of producing a triple gene-silenced culture. The resulting transfected culture (PV) was not silenced in the *EhCP-5* gene but remained silenced, as in the RBV culture, in both the *Ehap-a* and the *Ehlgl1* genes (unpublished data). To try to explain this failure, we examined whether a preference exists to use the promoter of the second gene, which was already silenced, to promote the silencing of a third gene. A plasmid in which the ORF of the *EhCP-5* gene was cloned under the control of the *Ehlgl1* 5′ promoter region, pSV (unpublished data), was tested for its ability to cause silencing of the *EhCP-5* gene when transfected into the plasmidless RBV trophozoites. Unfortunately, pSV did not silence the expression of *EhCP-5* (unpublished data). Other attempts to silence more than two genes in the same trophozoite are in progress.

## Discussion

We previously reported the silencing of expression of the gene that encodes amoebapore A, *Ehap-a,* in trophozoites of E. histolytica [12,21,44,51]. The gene-silenced clone G3) was created by transfection of the parent strain (HM-1:IMSS) with a hybrid plasmid (psAP-2) containing a 473 bp fragment of the 5′ regulatory region of *Ehap-a*. This fragment included a 140 bp segment of the 5′ end of an adjacent SINE1 retroposon element. Plasmids in which the sequences of the SINE1 element were omitted did not induce gene silencing [21]. Gene silencing was locked at the transcriptional level (TGS) and was inherited in the progeny cells even after removal of the plasmid. Silencing appeared to be irreversible, and attempts to reactivate the transcription of *Ehap-a* by cultivation of silenced trophozoites in the presence of inhibitors of DNA methylation or histone deacetylation have failed [12].

In our present work we have succeeded in silencing additional genes of interest: the light subunit of the Gal/GalNAc-inhibitable lectin and cysteine proteinase 5, both important virulence factors of *E. histolytica.* Silencing of the second gene was successful only under certain conditions.

First, the successful transfecting plasmids had the ORF of the second gene placed under the above-mentioned 5′ regulatory sequences of the *Ehap-a* gene, as demonstrated by plasmids pB33 or pAP-CP5.

Second, the plasmidless clone G3 trophozoites, which are already silenced in *Ehap-a,* were the only type that could be silenced at a second gene [12]. Transfections of strain HM-1:IMSS with the same plasmids resulted in overexpression of the gene instead of silencing. Another trophozoite culture that failed to silence a second gene was the plasmidless clone F1, which was also silenced in the *Ehap-a* gene. In contrast to clone G3, clone F1 trophozoites were obtained by transfection of HM-1:IMSS with a plasmid called psAP-1, which contained, in addition to the 473 bp of the 5′ upstream region of *Ehap-a*, the ORF and the 3′ regulatory region of the gene [12].

Third, the gene to be silenced was placed directly under the promoter sequences of the *Ehap-a* gene, with no intervening sequences as in plasmids pB33 and pAP-CP5. In plasmid pAY, the ORF of the *Ehlgl1* gene was preceded by sequences of the *Ehap-a* signal peptide, and this prevented the silencing in trans of the chromosomal copy of *Ehlgl1;* only the episomal gene copy was silenced. The inabilities of F1 trophozoites and pAY to silence a second gene, and the failure in RBV trophozoites to silence a third gene *(EhCP-5)* indicate that only G3 trophozoites (silenced in *Ehap-a* by transfection with a plasmid containing only the 473 bp 5′ upstream regions of *Ehap-a*) are capable of silencing additional genes that are ligated downstream, and of transferring the silencing to the chromosomal copy in trans.

Finally, both the SINE1 truncated sequences and the proximal 5′ regulatory sequences of *Ehap-a* must be present in the plasmid, upstream to the ORF of the second gene to be silenced. Omission of the SINE1 sequences, as in plasmid pP10, or replacement of the 5′ regulatory region with that of the *Ehlgl1* gene, as in plasmid pSG, resulted in expression of both the transgene and the chromosomal copy.

Our current working hypothesis on the gene silencing mechanism of E. histolytica is based on the proposed RNA-DNA recognition model of Grewal and Moazed [31]: Small heterochromatic RNA molecules may form an RNA-DNA triplex which then enables the binding of a silencing complex. Another example for the possible role of noncoding single-stranded RNA molecules in regulating gene expression was recently discovered in *Drosophila* [52]. We have shown that nuclei of gene-silenced E. histolytica trophozoites contain short single-stranded RNA molecules with homology to the 5′ end of the SINE1 element upstream to the *Ehap-a* gene [21]. These single-stranded RNA molecules may serve as the small heterochromatic RNA molecules that bind to the DNA and form a triplex, as proposed in the models [31,52]. The composition of the silencing complex that binds to the triplex is not yet well defined, but it appears to consist of DNA-binding proteins and enzymes that modify the histone residues and result in the conversion of euchromatin to heterochromatin. This chromatin modification then extends or spreads, in cis, downstream until it reaches a boundary region or an insulator element with barrier activity that prevents condensed heterochromatin from extending into adjacent euchromatin domains carrying transcriptionally active genes [53]. We have shown that the transcription of an uncharacterized gene (TIGR genome database locus 88.m00160) located downstream of *Ehap-a* as well as the SINE1 element upstream to *Ehap-a* were not silenced [21]. On the other hand, we have found that in addition to the *Ehap-a* gene, two other genes, *Ehap-b* and the gene encoding *SAPLIP1*, were silenced. These two genes have considerable homology and are on the same branch of the phylogenetic tree as the *Ehap-a* gene [45,46], but according to the E. histolytica genome they are located in other contigs and scaffolds. The homology between the genes was found in the ORF sequences but not in the regulatory regions of these genes, and there are no SINE1 elements adjacent to those genes. Other genes of these families (*Ehap-c* and *SAPLIPs 5* and *14*), which are located on other phylogenetic branches and have no significant homology to *Ehap-a* [46], were not silenced in G3 trophozoites. Moreover, we have found that the silencing of *Ehlgl1* in the RBV trophozoites was also accompanied by the down-regulation of the closely related and homologous *Ehlgl2* and *Ehlgl3* genes. These findings raise the possibility that the observed phenotype may not be solely due to silencing of the targeted gene but may be influenced by additional inadvertently down-regulated genes.

Two important questions remain. How does gene silencing spread from the episomal gene copy to the endogenous chromosomal gene? What are the biochemical markers of this process [43]? In the silenced clone G3 trophozoites, the *Ehap-a* gene domain was shown to become associated with heterochromatin, blocking its transcription [21]. Since epigenetic gene silencing was shown to continue in the progeny cells in the absence of the plasmid, a molecular mechanism dependent on sequence recognition must exist to enable the spread or extension of the heterochromatin to the genomic domain of the *Ehap-a* gene in the replicating DNA of dividing cells. It is thus reasonable to assume that when a plasmid containing the 473 bp 5′ upstream region of *Ehap-a* (including the SINE1 sequences) is reintroduced into such gene-silenced trophozoites, it generates short (140 nucleotide) single-stranded SINE RNA molecules that can bind to the 5′ region of the SINE DNA; this mediates the binding of a silencing complex upstream to the episomal *Ehap-a* gene promoter, as proposed in the model of Grewal and Moazed [31]. The silencing complex then begins to modify the euchromatin on the 5′ upstream region of the episomal *Ehap-a* gene, and heterochromatinization then spreads downstream, preventing the transcription of any genes that are ligated to it (e.g., *Ehlgl1* or *EhCP-5*). At this stage the transfected cells may have acquired the “memory” to modify the chromatin and silence the expression of the ORFs that are on the episomal copy. Heterochromatinization is then transferred, in trans, to the chromosomal gene by a mechanism which most likely uses a sequence-dependent marker. This in-trans spreading was shown to occur, however, only in transfectants of the already silenced clone G3 trophozoites. If the direct connection between the *Ehap-a* promoter and the second gene was interrupted by the insertion of 44 bp from the *Ehap-a* ORF (as in plasmid pAY) or by the entire ORF (as in the clone F1 silenced trophozoites), these insertions may have created an artificial boundary or introduced an insulator activity [53] that prevented the spread of the heterochromatin as well as the silencing of the second genomic gene in-trans. A similar process may explain our failure to silence a third gene in the double-silenced RBV trophozoites.

Our ability to sequentially silence the expression of other important genes could improve our understanding of the individual role that certain genes may have in the metabolism or pathogenesis of the parasite. G3 trophozoites, which are so far the only amoeba in which we can silence a second gene, possess a very low virulence and are incapable of killing mammalian cells; this could mask the potential contribution of other virulence factors. On the other hand, both of the double-silenced trophozoites, RBV and RB8, have additional deficiencies in critical virulence factors, which should lower their virulence even more. Thus it will be interesting to examine the responses and pathogenicity of RB8 trophozoites, which do not express AP-A and CP-5, and RBV trophozoites, which do not express Lgl1 and AP-A proteins. Studies of these E. histolytica strains in the Hu-mice intestinal engrafted model will help us better understand the contribution of CP-5 and Lgl1 proteins to the inflammatory process and the development of colitis [38,39,44]. In parallel we are also exploring silencing of a gene not involved in virulence by searching E. histolytica genome databases (http://www.tigr.org/tdb/e2k1/eha1 and http://www.sanger.ac.uk/Projects/E_histolytica) for genes located near other SINE elements; the goal is to produce a silenced trophozoite that is virulent and possibly useful for the subsequent silencing of a second gene.

Stable, double gene-silenced amoebae may also be useful for immunoprotection against oral infection by the virulent parasite. The double gene-silenced amoebae are virulence-attenuated, yet they retain the original antigenic repertoire of virulent HM-1:IMSS trophozoites; thus future investigations will focus on whether these harmless trophozoites can colonize the colon (in a primate model) and evoke a significant, effective, and long-lasting immunoprotective response.

## Materials and Methods

### Strain and culture conditions.

Trophozoites of E. histolytica strain HM-1:IMSS and of the plasmidless gene-silenced clone G3 [12] were grown at 37 °C in TYI-S-33 medium [54]. Transfection of trophozoites was performed as previously described [55], and cultures were grown in the presence of the neomycin derivative G418. Plasmids were removed by growing transfectants without G418 for 3 wk, then cloning trophozoites and testing for cultures that were devoid of plasmid by PCR amplification of the gene encoding neomycin resistance in genomic DNA [12]

### Plasmid constructs.

The pEhActNeo shuttle vector, which served as the basic construct, contains the *Neo* gene, which confers resistance to G418, flanked by the 5′ and 3′ regulatory sequences of the amoeba actin 1 gene *Ehactin* [56–58] and the E. histolytica autonomous replication sequence, both cloned in pBluescript II SK (−). Primers used for the construction of the different cassettes are listed in Table 2.

**Table 2:**
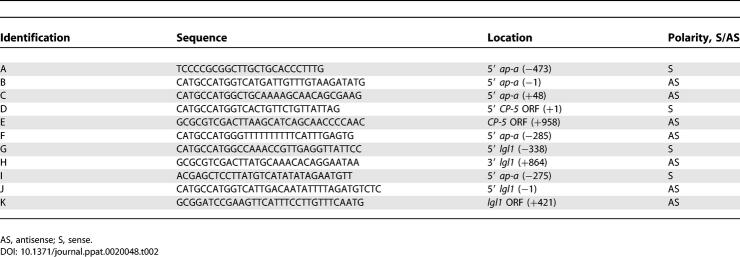
Primers Used for Plasmid Constructs

Plasmid pB33 includes the 5′ upstream segment (473 bp) of the *Ehap-a* gene amplified by PCR from psAP-1 [12] using primers A and B (Table 2). The ORF region of the *Ehlgl1* gene and the 3′ *Ehactin* flanking region were prepared by digestion of the intermediate plasmid construct pSA21 with NcoI/BamHI [35,47]. This plasmid is based on pBluescript IIKS and includes a cassette containing the 5′ flanking region of the *Ehactin* gene, the ORF of the *Ehlgl1* (867bp) gene, and the 3′ flanking region of *Ehactin*.

To construct plasmid pAY we first generated, by PCR with plasmid psAP-1 [12] as a template and primers A and C (Table 2), a 521 bp fragment that included the 473 bp of the 5′ upstream region of the *Ehap-a* gene and 44 bp of the signal peptide of the *Ehap-a* gene. This fragment was then ligated to the segment containing the ORF of *Ehlgl1* with the 3′ *Ehactin* flanking region as above.

The construction of pAP-CP5 was similar to that of pB33. The 473 bp 5′ upstream of the *Ehap-a* gene was generated by PCR using primers A and B (Table 2) on plasmid psAP-1 [12]. The ORF of *EhCP-5* [41,42] was generated by PCR using primers D and E (Table 2) on genomic DNA of strain HM-1:IMSS, and the 3′ flanking region of *Ehactin* was generated by digestion of plasmid pSA21 [47] with SalI/BamHI.

For plasmid pSG, a DNA fragment containing 188 bp of the 5′ upstream region of *Ehap-a* consisting of the truncated SINE1 sequence and the T-rich region was amplified by PCR from plasmid psAP-1 [12] using primers A and F (Table 2). The resulting 188 bp fragment was ligated to the 5′ end of another segment (1,203 bp) consisting of the *Ehlgl1* ORF and 338 bp of the *Ehlgl1* 5′ upstream region. This latter fragment was generated by PCR amplification of genomic DNA of strain HM-1:IMSS using primers G and H (Table 2) followed by ligation to the 3′ *Ehactin* regulatory segment.

Plasmid pP10 was constructed from a DNA fragment of 275 bp of the 5′ upstream region of the *Ehap-a* gene generated by PCR from plasmid psAP-1 [12] using primers I and B (Table 2) and ligated to the ORF of *Ehlgl1* with the 3′ actin flanking region, as in pB33.

For plasmid pTL, the 473 bp fragment of the 5′ upstream region of *Ehap-a* and a fragment of 421 bp starting from the 5′ end of the ORF of *Ehlgl1* (truncated *Ehlgl1*) (894 bp) were generated by PCR from plasmid pB33 using primers A and K (Table 2)

Plasmid pSV contains a 338 bp fragment from the 5′ upstream region of *Ehlgl1* generated by PCR from genomic DNA using primers J and G. This fragment was ligated to the ORF of the *EhCP-5* gene and then to the 3′ flanking region of the *Ehactin* gene.

Each of the cassettes mentioned above was then ligated to the digested and dephosphorylated pEhActNeo shuttle vector as previously described [12,21]

### Northern blots and RT-PCR.

Total RNA was prepared using the RNA isolation kit TRI Reagent according to the manufacturers protocol (Sigma, St. Louis, Missouri, United States). RNA was size-fractionated on a 4% polyacrylamide denaturing gel containing 8 M urea, and subsequently blotted to a nylon membrane. Using stringent conditions, hybridization was carried out with different probes randomly labeled using the Redi-Prime II kit (Amersham Life Science, Bucks, United Kingdom) and washed with 0.1% SDS, 0.1× SSC (1× SSC is 0.15 M NaCl plus 0.015 M sodium citrate). For RT-PCR, RNA samples were treated with RNase-free DNase (Promega, Madison, Wisconsin, United States) and after phenolation and sedimentation, 2 μg of RNA were reacted with AMV reverse transcriptase (Promega) according to the manufacturer's protocol using an oligo dT-adaptor as primer (Table 1). The cDNA product was diluted five times and used for the subsequent PCR reactions.

### Detection of cysteine proteinases.

The cysteine proteinase inhibitor Fmoc-Tyr-Ala-diazomethylketone (Bachem, Bubendorf, Switzerland), which covalently binds to cysteine proteinases [49], was labeled at its tyrosine residue with I^125^ as previously described using the iodogen protocol (Pierce, Rockford, Illinois, United States). The [I^125^]-labeled inhibitor (10 μCi, 10 μg/ml) was added to logarithmically growing cultures of trophozoites (5 × 10^4^/ml) for 18 h. The trophozoites were then harvested, washed, and lysed in the presence of protease inhibitors [50]. Bands containing the covalently bound [I^125^]-labeled inhibitor were detected after SDS-PAGE separation on reducing gels and overnight exposure of the dried gel to X-ray film.

### Cysteine proteinase activity.

Proteinase activity of trophozoite lysates was determined spectrophotometrically by the rate of degradation of the cysteine proteinase fluorogenic substrate Z-Arg-Arg-pNA (Bachem) in the presence or absence of DTT (20 mM) as described [40].

### Gal/GalNAc-lectin capping assay.

Capping of the Gal-lectin surface molecules was performed essentially as previously described [35]. Freshly harvested trophozoites were washed in PBS and divided into two tubes each (~2 × 10^6^ per tube). To each tube two monoclonal antibodies (3F4 and 7F4; a gift from Dr. Richard Vines, TechLab, Blacksburg, Virginia, United States) against the heavy (170 kDa) subunit of the Gal-lectin [34] were added at 1:30 dilution. One of the tubes was kept at 4 °C and the other at 37 °C, to induce capping, for 20 min. Fixation of trophozoites was performed with the addition of paraformaldehyde to a final concentration of 3.7% for 15 min, followed by a wash with 50 mM NH_4_Cl in order to block free aldehydes. Final blocking was with 2% fetal calf serum. After washing, the fixed trophozoites were incubated with FITC-labeled goat anti-mouse antibodies (Jackson Immuno Research, West Grove, Pennsylvania, United States) at a 1:200 dilution. Finally, samples were viewed with a confocal microscope (Fluoview FV500; Olympus, Tokyo, Japan).

### Liver lesions in hamsters.

Amoebic liver abscesses in Syrian golden hamsters were generated by laparotomy and injection of freshly harvested trophozoites (5 × 10^5^) directly into the frontal liver lobe as previously described [35]. Hamsters were sacrificed after 1 wk and the hepatic lesions examined.

## Supporting Information

### Accession Numbers

The GenBank (http://www.ncbi.nlm.nih.gov) accession numbers of the E. histolytica genes mentioned in the text are *ap-a* (X70851), *ap-b* (X76904.1), *ap-c* (X76903.1), *cp-5* (X91644.2), *lgl-1* (M96024), *lgl-2* (L20898), and *lgl-3* (U32617); and genes encoding SAPLIP 1 (AZ529784), SAPLIP 5 (AZ530711), and SAPLIP 14 (AZ690015).

## Abbreviations

AP-A: amoebapore A
CP-5: cysteine proteinase 5
LINE: long interspersed nuclear element
ORF: open reading frame
PTGS: posttranscriptional gene silencing
SINE: short interspersed nuclear element
TGS: transcriptional gene silencing
UTR: untranslated region
